# Reviewing the Significance of Vitamin D Substitution in Monoclonal Gammopathies

**DOI:** 10.3390/ijms22094922

**Published:** 2021-05-06

**Authors:** Vanessa Innao, Alessandro Allegra, Lia Ginaldi, Giovanni Pioggia, Massimo De Martinis, Caterina Musolino, Sebastiano Gangemi

**Affiliations:** 1 Department of Human Pathology in Adulthood and Childhood “Gaetano Barresi”, Division of Haematology, University of Messina, 98125 Messina, Italy; vinnao@unime.it (V.I.); cmusolino@unime.it (C.M.); 2 Department of Life, Health and Environmental Sciences, University of L’Aquila, 67100 L’Aquila, Italy; lia.ginaldi@univaq.it (L.G.); demartinis@cc.univaq.it (M.D.M.); 3 Allergy and Clinical Immunology Unit, Center for the Diagnosis and Treatment of Osteoporosis, AUSL 04 Teramo, 64100 Teramo, Italy; 4 Institute for Biomedical Research and Innovation (IRIB), National Research Council of Italy (CNR), 98164 Messina, Italy; giovanni.pioggia@cnr.it; 5 Department of Clinical and Experimental Medicine, School and Operative Unit of Allergy and Clinical Immunology, University of Messina, 98125 Messina, Italy; gangemis@unime.it

**Keywords:** multiple myeloma, MGUS, smoldering multiple myeloma, vitamin D, cancer, immune response, anti-myeloma therapy

## Abstract

Vitamin D is a steroid hormone that is essential for bone mineral metabolism and it has several other effects in the body, including anti-cancer actions. Vitamin D causes a reduction in cell growth by interrupting the cell cycle. Moreover, the active form of vitamin D, i.e., 1,25-dihydroxyvitamin D, exerts various effects via its interaction with the vitamin D receptor on the innate and adaptive immune system, which could be relevant in the onset of tumors. Multiple myeloma is a treatable but incurable malignancy characterized by the growth of clonal plasma cells in protective niches in the bone marrow. In patients affected by multiple myeloma, vitamin D deficiency is commonly correlated with an advanced stage of the disease, greater risk of progression, the development of pathological fractures, and a worse prognosis. Changes in the vitamin D receptor often contribute to the occurrence and progress of deficiencies, which can be overcome by supplementation with vitamin D or analogues. However, in spite of the findings available in the literature, there is no clear standard of care and clinical practice varies. Further research is needed to better understand how vitamin D influences outcomes in patients with monoclonal gammopathies.

## 1. Introduction

Vitamin D (Vit D), the so-called sunshine vitamin, plays crucial roles in many physiological functions, and vitamin D deficiency is associated with many acute and chronic pathologies, including disorders of calcium metabolism, autoimmune, cardiovascular and infectious diseases, diabetes and some cancers [1]. The term vitamin D actually identifies a group of molecules (pro-hormones), mainly present in the form of ergocalciferol (vitamin D2) and cholecalciferol (vitamin D3). Endogenous vitamin D levels change greatly during people’s lifetime in response to diet or latitude exposition. Exposure of human skin to solar UVB radiation leads to the conversion of 7-dehydrocholesterol to D3 in the skin. Vitamin D3 is a prohormone that is activated by sequential hydroxylations, both at the systemic (liver and kidney) and local (skin) levels, to produce the biologically active form [2]. Other sources of vitamin D are milk and dairy products. Both D3 made in the skin and dietary D2 can be stored in fat cells and then released into the blood and transported to the liver, where they are converted by the vitamin D-25-hydroxylase to 25-hydroxyvitamin D [25(OH)D], which represents the major circulating form of typically measured vitamin D. It is biologically inactive and must be further converted in the kidneys by 25-hydroxyvitamin D-1α-hydroxylase to its biologically active form 1,25-dihydroxyvitamin D [1,25(OH)2D], which binds to a specific cellular receptor and can thus carry out its action, linked not only to bone metabolism, but also to other physiological functions. Several hydroxylases and other enzymes, also expressed extrarenally in a multitude of tissues, are involved in the metabolism of vitamin D, and most vitamin D metabolites have been found to display biological activities [3].

1,25(OH)2D functions more like a steroid hormone than a vitamin, entering cells and binding to a nuclear receptor that stimulates the production of various proteins, especially calcium transporters. Activated vitamin D receptor (VDR) interacts with retinoid X receptor (RXR) and forms a VDR/RXR/cofactor complex, which binds to vitamin D response elements in the promoter region of target genes to regulate gene transcription. A membrane-bound VDR may also exist and mediate non-genomic actions of 1,25(OH)2D. The VDR-induced rapid responses via non-genomic membrane-associated mechanisms involve an alternative ligand-binding site (A-pocket). Vitamin D hydroxyderivatives exhibit different affinities for multiple receptor targets and regulate different biological functions through the modulation of distinct receptor signaling pathways [1].

From a metabolic point of view, 1,25(OH)2D promotes calcium reabsorption in the kidney, intestinal absorption of both phosphorus and calcium, bone mineralization and the differentiation of cells involved in bone remodeling.

The VitD-Parathormon (PTH)-calcitonin endocrine system plays important roles in the adaptation to variations in dietary calcium and phosphorus intakes. 1,25(OH)2D regulates intestinal calcium and phosphate absorption, providing the substrates for bone mineralization. Unlike VitD, calcitonin decreases plasma calcium levels by promoting urinary elimination and its deposition in bone, while parathyroid hormone inhibits renal reabsorption of phosphates, increases calcium reabsorption, stimulates the kidney to produce the active metabolite of VitD, and promotes the release of calcium from the bone. The production of PTH, calcitonin and 1,25(OH)2D and vitamin D are strictly dependent on the plasma concentration of calcium. Hypocalcemia stimulates the production of PTH and 1,25(OH)2D, while an increase in plasma calcium levels favors the synthesis of calcitonin. Proper regulation of bone mineralization processes depends on the functioning of this delicate balance. Therefore, the main role of 1,25(OH)2D is to promote bone calcification by increasing the absorption of calcium from the diet, meaning it is a central regulator of mineral homeostasis, calcium metabolism and bone tissue.

However, there is also a direct physiological action of 1,25(OH)2D in the bone remodeling cycle. This is carried out by specialized cells in the bone, the differentiation of which is promoted by 1,25(OH)2D [2]. Bone physiology depends on the activity of the three main types of cells, as well as interaction with the hematopoietic system in bone marrow: Osteoblasts, derived from mesenchymal progenitors, are bone-forming cells; osteocytes, which have a mesenchymal origin, are embedded in the mineral matrix, and contribute to matrix deposition and mineralization; and osteoclasts are the cells responsible for bone resorption and have a hematopoietic origin. VitD directly acts on osteoblasts, activating osteoclastogenesis and bone resorption. This metabolic pathway for the activation of bone resorption depends on the expression of VDR by osteoblasts. 1,25(OH)2D is recognized by its receptor in osteoblasts, causing an increase in the expression of receptor activator of NFκB ligand (RANKL), which binds to RANK expressed on the surface of osteoclast progenitor cells, inducing osteoclast maturation. The mature osteoclast removes calcium and phosphorus from the bone to maintain blood calcium and phosphorus levels. Adequate calcium and phosphorus levels promote the mineralization of the skeleton [3]. The expression of many key genes for osteoblast maturation and mineral deposition is also modulated by VitD, mostly by increasing the gene expression of proteins essential for bone homeostasis such as type I collagen, alkaline phosphatase, osteopontin and osteocalcin [2]. In addition to stimulating osteoblast maturation, 1,25(OH)2D regulates skeletal hormone expression. It suppresses PTH and increases the expression of the osteocyte-specific hormone fibroblast growth factor 23 (FGF23), which is a marker of osteocytes in the early phase of secondary mineralization [3]. Furthermore, in vitro studies have shown activity of the VitD-activating enzyme 1alpha hydroxylase in bone cells, suggesting reciprocal interactions between VitD and skeletal cells [4].

Several studies have reported the role of vitamin D in the modulation of cancer risk, revealing that deficiency is correlated with pro-inflammatory responses [5,6,7]. Thus, in this review, we aim to assess the benefits and disadvantages of vitD in monoclonal gammopathies, and to explore the possible role of vitamin D, combined with standardized therapies, in the management of gammopathies.

Multiple myeloma (MM) is an aggressive malignancy determined by the clonal proliferation of abnormal plasma cells, and it represents the most frequent hematologic neoplasm in patients over 65 years old [8,9]. MM accounts for 1–1.8% of all cancers with an estimated occurrence in Europe of 4.5–6.0 in every 100,000 people per year [10]. It is clinically defined by augmented bone marrow (BM) plasmacytosis, serum and/or urine monoclonal immunoglobulin, secretion of free light chains, hypercalcemia, renal insufficiency, anemia, and bone pain due to osteolytic disease [11,12,13]. MM also stimulates osteoclasts in the bones via the nuclear factor kappa-B ligand (RANKL), resulting in the destruction of bone via lytic lesions that cause pain, fractures, mobility issues and calcinosis. The hallmark end-organ damage of MM is referred to as “CRAB” symptoms: hypercalcemia, renal involvement, anemia, and bone lesions [14].

Survival rates have improved over the last 10 years using novel agents such as triplets of several generations of proteasome inhibitors (PIs) and immunomodulatory drugs (IMiDs), mono- and bi-clonal antibodies (moAbs and BiTEs), new alkylating agents (Melflufen), chimeric antigen receptor (CAR) T-cell therapy, and promising experimental approaches based on vaccine therapy or antisense microRNA (AntagomiRs). However, MM relapse has been reported in a large number of patients [15,16,17,18,19,20,21,22,23]. Several studies have been conducted to identify the best prognostic predictors in patients with MM, revealing that minimal residual disease is the best marker today [24,25]. 

A major scientific and clinical challenge in this disease is finding a balance between myeloma cell killing efficacy and toxicity for patients. Considering the role of immunity in MM, a deeper understanding of the interaction between neoplastic plasma cells and the BM immunome is of the utmost importance for the development of more effective and safe treatments.

In a neoplastic subset of patients, most of whom suffered symptomatic multiple myeloma (MM), VitD deficiency was frequently related to a late stage of the disease, reduced survival, and greater risk of the progression and development of pathological fractures in asymptomatic patients [26]. Pleiomorphisms of the VDR often participate in the development of deficiency states [27], which can be overcome by supplementation with VitD or analogues. In addition, VitD has been found to improve osteoblastic differentiation and maturation, which also reduces the lineage switching of MM plasma cells through an osteoclast-like transformation. However, despite the findings available in the literature, there are no guidelines for the use of VitD as an adjuvant in the treatment of multiple myeloma patients. 

As the number of studies on the viral infection SARS-CoV2 has increased recently, the role of VitD status in immune responses has returned to the forefront of research, revealing worse outcomes in VitD-deficient patients [28,29,30,31].

## 2. Vitamin D and the Immune System

The binding of the active metabolite of vitD (1,25(OH)_2_D_3_ or calcitriol) with its nuclear receptor, the VDR, which forms a heterodimeric complex with the retinoic acid receptor and interacts with transcript factors, results in the regulation of hundreds of different genes [32,33], increasing the intestinal and kidney absorption of calcium. A deficiency in vitD results in secondary hyperparathyroidism, causing increased osteoclast activity, osteopenia, and osteoporosis [34]. VitD is also involved in proliferation and cell differentiation processes, as well as immune modulation [35]. In the immune system, for example, it promotes the differentiation of monocytes [36] and inhibits lymphocyte proliferation through an increase in cytokines, such as IL-2, IL12, and interferon-gamma. In addition, VitD suppresses immune responses mediated by Th1 cells, induces the proliferation of T-reg cells, inhibits Th17-cells (IL-17-producing T cells), increases the Th2 population, and inhibits the growth and functioning of natural killer (NK) and dendritic cells [37,38,39,40,41]. These effects are the inverse of the effects of MM progression, because elevated levels of IL-17, which is produced by Th17 cells, promotes MM cell proliferation and the development of skeletal-related events (SREs) [42,43]. These effects are also amplified by a reduction in myeloid-derived suppressor cells (MDSCs), which are promoted by VitD and have an effect on macrophage activity.

A population of suppressive CD11b+Gr-1+ cells was designated as MDSCs, which are a unique category of the myeloid lineage that prevents the development of cytotoxic T lymphocytes (CTLs) in vitro and induces antigen-specific CD8+ T-cell tolerance in vivo. MDSC have several functions that permit tumor survival, including the ability to promote angiogenesis [44]. There is growing evidence that VitD signaling may be a regulator of MDSC biology. The earliest of these studies examined the impact of VitD on CD34+ cells that are precursors for MDSC [45]. The active form of VitD, 1,25 dihydroxyvitamin D, can block the development of the immunosuppressive function in cultured CD34+ BM cells [46]. Consistent with these actions, 1,25(OH)2D treatment in mice with 4-NQO-induced squamous cell carcinoma decreased invasive cancer, reduced tumor MDSC number, blocked IL-6 induced recruitment of MDSC, and reduced the T-cell suppressive capacity of MDSC from the tumor [47]. It has been demonstrated that 1,25(OH)2D may interfere with the production of tumor-derived signals that promote MDSC differentiation such as GM-CSF, IL-6, and miR155-containing exosomes [48]. Finally, 1,25(OH)2D signaling through the VDR reduces the immunosuppressive capability of MDSC [49].

**Figure 1:**
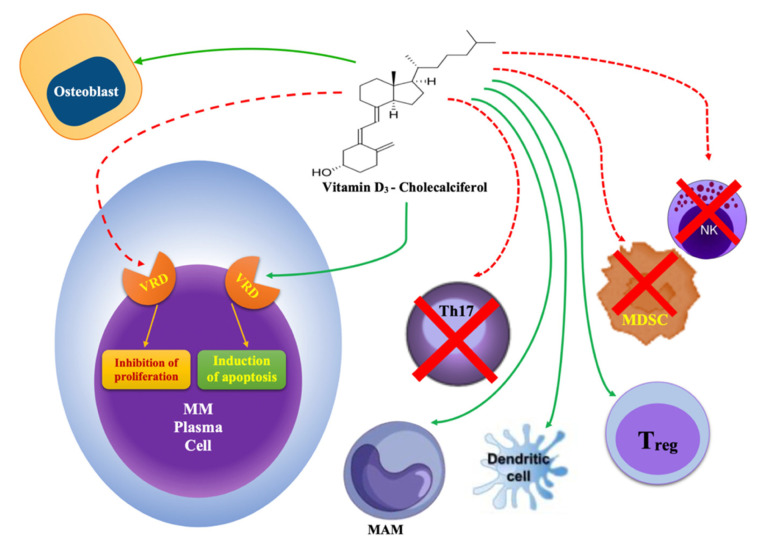
Vitamin D actions on the cell compartment of the bone marrow microenvironment. Green arrows indicate the stimulating effects on expansion and function, while the red dashed arrows indicate the inhibition of proliferation and cell functions [35,36,37,38,39,40,41,42,43,44,45,46,47,48,49,50,51,52,53,54].

Macrophages are phenotypically and functionally heterogeneous cells. They exert a multitude of biological activities conditioned both by tissue microenvironment stimulation and cytokines signals [50]. M1 and M2 macrophages exert opposite activities, i.e., anti-inflammatory versus proinflammatory responses, immunogenic versus tolerogenic activities, and tissue repair versus tissue destruction, respectively [51]. Macrophages are a vital component of the tumor microenvironment and are crucial mediators of tumor progression. Accumulating evidence demonstrates that macrophage infiltration is associated with poor overall survival in MM. Indeed, macrophages influence numerous pathways critical for the initiation and progression of MM including homing of malignant cells to BM, tumor cell growth and survival, drug resistance, angiogenesis, and immune suppression [52]. 1α,25(OH)_2_D_3_ affects macrophage polarization towards the M2 phenotype [53]. Studies showed that VitD, by acting through its receptors, upregulates transcription of the anti-inflammatory dual-specificity protein phosphatase 1 (DUSP1) gene, which down-regulates the expression of inflammatory chemokine IL-8 produced by over-reactive (hyperinflammatory) macrophages (Figure 1) [54]. Thus, vitamin D is proposed as a promising therapeutic approach for overcoming resistance to immunomodulating therapies [55,56,57,58].

Innate and adaptative immune cells modulate the local VitD concentration by converting 25(OH)D_3_ into 1,25(OH)2D_3_, resulting in the suppression of pro-inflammatory cytokines and the alteration of the function and composition of skin and gut microbiota [59]. VitD deficiency is correlated with an increased inflammatory response, the development of diseases, and risk progression in autoimmune diseases, psoriasis-associated osteoporosis, and multiple sclerosis [60,61,62]. It has also been demonstrated to increase the risk of cancer. In some types of cancer cells, VitD has exhibited antiproliferative activity.

VDR expression is also relevant. It is almost ubiquitous in human tissues including monocytes, T-lymphocytes, and cancer cells. In fact, in addition to its key role of maintaining skeletal homeostasis, this compound has shown a strong ability to interact with the immune system [63,64] and to regulate susceptibility to autoimmune diseases (e.g., inflammatory bowel disease and type-1 mellitus diabetes) and cancer development, owing to its regulation of the expression of several tumor-related genes and the inhibition of angiogenesis, cancer cell growth, adhesion and metastatization [65,66,67,68]. 

### Vitamin D and Cancers 

In recent years, the role of VitD deficiency as a predictor of poor overall survival has been widely demonstrated in oncological patients diagnosed both with solid and hematological malignancies. The confinement associated with the global pandemic has certainly played a key role in determining the serum VitD levels of patients with COVID-19 infection and solid neoplasms, such as colorectal, breast, lung, ovarian, bladder, prostate, and thyroid cancer. VitD levels, in addition to VRD polymorphisms, have proven to impact the outcomes of these patients [35,69,70,71,72,73,74]. However, studies have found contrasting results. Most have revealed that higher serum 1,25(OH)_2_D_3_ levels are inversely correlated with cancer risk [75,76,77], which supports a potential anti-cancer effect of replacement therapy, with improved survival in patients treated with exogenous analogues [78]. A high number of patients with hematological malignancies show low VitD levels of between 30% and 80%, regardless of the type of malignancy (myeloid or lymphoid cancers) and the treatment received [79,80,81,82,83], showing that those with higher vitamin levels have a better chance of survival [84,85,86]. Interestingly, VitD has an anti-proliferative action on activated B-cells and plasma cells, despite the increase in the expression of CD38; however, this is not followed by an increased CD27 expression caused by VitD exposition [87]. EB1089, a 1, 25-dihydrxyvitamin D(3) analog, inhibits MM cell growth, thus inducing cell cycle arrest by the direct stimulation of caspase 3 protease and downregulation of the anti-apoptotic Bcl-2 protein [88,89,90]. 

## 3. Vitamin D Status in Multiple Myeloma

Among hematological malignancies, MM is most closely related to calcium metabolism and SREs due to the associated osteoclast (OC) activation and osteoblast paralysis resulting from the interaction between myeloma cells and the bone microenvironment [91]. MM-induced bone lesions (myeloma bone diseases—MBDs) are a frequent complication, both at the onset and during the natural history of myeloma, which is correlated with a significant worsening of patient quality-of-life [92]. 

The negative impact of VitD deficiency has been demonstrated in plasma cell neoplasms, with a direct correlation with low serum VitD levels—a late disease stage according to the International Staging System (ISS)—and higher serum C-reactive protein in a large cohort of patients [93,94]. Similarly, in 2015, researchers reported that increased plasma cells in BM are correlated with VitD levels < 10 ng mL^–1^ [95]. In MM patients, most of whom lack vitamin D [96], this correlation was also valid in a long-term follow-up study, where the risk of progression, development of MBD and osteoporosis, and all-cause mortality was low in patients with adequate VitD levels (>75 nmol L^−1^) [97]. Moreover, in these patients, VitD analogues have been shown to promote cell cycle arrest [88,98]. 

Following the rational treatment of all patients with VitD supplements, doubts about the dosage to be used to induce toxic effects on myeloma cells and to stimulate immune response have arisen [99]. We already know that a high serum concentration of 1,25(OH)_2_D_3_ may cause conditions such as hypercalcemia, through a clinical sign that is often present in myeloma patients with massive skeletal involvement. Thus, studies on alternative analogues (ZK168281) as therapeutic options in VitD-induced hypercalcemia are ongoing [100,101]. However, on the basis of laboratory data, some studies have revealed that VitD-deficient patients with hyperparathyroidism and hypercalcemia that were treated with oral calciferol do not experience worsening calcium levels, and that they are instead stabilized or decreased, while there is a reduction in parathormone levels [102,103].

Some studies have observed that MM cells may cause direct erosive damage to bone tissue due to an osteoclast-like transformation, expressing typical OC markers [104,105]. The expression of several transcription factors, such as PAX 5, MEF2C (Myocyte Enhancer Factor 2C), CCAAT/C/EBPa (CCAAT/Enhancer Binding Protein a), and HDAC-7 (Histone Deacetilase 7), determines this lineage switch [106,107,108,109]. Non-toxic doses of 1,25-dihydroxyvitamin D3 have been shown to induce this trans-differentiation of MM cells into monocytoid precursors, thus promoting their OC-like activity [110,111]. 

VitD deficiency stimulates osteoclast-mediated bone resorption and reduced bone mineralization via TNF-related activation-induced cytokines (TRANCE) and osteoprotegrin (OPG) [34]. In addition, several intracellular signaling pathways, including RANK/RANKL/OPG, Notch and Wnt, are involved in the persistent, dynamic, and progressive bone loss of these patients. In combination with anti-myeloma drugs, these pathways are used as targets of many promising therapeutic approaches in the management of MBDs, including some that have already been approved, such as bisphosphonates and denosumab (monoclonal antibody against the osteoclast activator, RANK-Ligand) [112], and others that are still being studied [113]. In addition, it is well known that MM cells express VRD, which activates at nanomolar concentrations of 1,25-dydroxy vitamin D. However, only supra-physiological amounts of 1,25D3 resulting in an antiproliferative effect [114]. Moreover, in MM cell lines, proapoptotic action also occurs due to the analogue EB1089 in the presence of IL-6, with synergistic activity and dexamethasone [89]. In 2004, a study reported similar results of treating MM cell lines with combinations of dexamethasone, all-trans retinoic acid (ATRA), 1,25-dihydroxyvitamin D, and interferon-alpha. Studying single groups and comparing them with controls, high rates of apoptotic cells in all studied populations were observed [115].

As reported by other researchers, VRD upregulation was observed in osteoblast precursors of MM patients treated with bortezomib (BTZ), revealing a large spectrum of anti-myeloma action in this proteasome inhibitor, both against MM plasma cells and BM microenvironments, and supporting the usefulness of supplementation with 25-dihydroxycholecalciferol in bortezomib-based regimens [116]. After all, PIs have shown better effectiveness in patients with MM and bone lesions [117]. Another advantage of VitD supplementation is that low serum vitamin D levels are correlated with the most severe neuropathy in patients treated with bortezomib and thalidomide [118]. 

The role played by VitD, in combination with lenalidomide, in inducing a response to novel anti-CD38 Felzartamab (MOR202) was reported in Bush et al. showed that VitD regulates myeloma-associated macrophage (MAM) activity, thus increasing vitamin D-1-hydroxylase CYP27B1 levels in these cells in vitro and resulting in an improved tumoricidal activity of MOR202 against MM cells ex vivo [119]. CYP27B1, together with VDR, is also expressed in other plasmablastic B-cell malignancies, such as plasmablastic lymphoma and diffuse large B-cell lymphoma. In these malignancies, VitD was shown to inhibit malignant plasmablastic cell proliferation, depending on MYC protein inhibition, and it was found that synthetic ROR ligand SR-1078 can enhance the antiproliferative effect of the vitamin itself [120]. 

VRD polymorphisms have been reported in MM cell lines with an increased risk stage. Specifically, single-nucleotide polymorphisms (SNPs) within vascular endothelial growth factor (VEGF) and VDR (Fok1) have been reported in several studies [121,122,123,124]. These reports were confirmed by a different group of researchers, who studied plasmablastic lymphoma and myeloma cells more broadly [120]. It was found that the higher frequency of VDR gene polymorphisms reported in MM patients is correlated with a higher MM development risk, thus supporting the active role of VitD in these patients. 

The validity of oral calcitriol as an active compound in supporting therapy after autologous stem cell transplantation (ASCT) was demonstrated by a previous study with high statistical sensitivity. By administrating 0.25 mg three times daily from transplantation to day 30, the study showed a better recovery of the absolute lymphocyte count (ALC) and relapse-free survival (RFS) in the experimental group compared to the placebo control, thus confirming the active role of VitD in the immune response. In a median of 29 months follow-up, relapse-free survival was significantly better in the calcitriol group (77.0%, SE  =  7.0% vs. 59.0%, SE  =  8.0%; *p*  =  0.03). Moreover, the median time to absolute lymphocyte count recovery was significantly shorter in the calcitriol group (≥0.5 × 10^3^/μL: 13 vs. 20 days; *p*  <  0.001), and absolute lymphocyte count recovery rates on day 15 (≥0.5 × 10^3^/μL: 82.1% vs. 42.5%; *p*  <  0.001) and on day 30 (≥1.0 × 10^3^/μL: 91.7% vs. 57.5%; *p*  =  0.001) were significantly higher with calcitriol [125]. 

An effect was also registered on hemopoiesis due to a reduction in the apoptosis of CD34+ cells and their increased number and activity [126]. In contrast to these results, two studies that included patients undergoing ASCT questioned the importance of vitD in improving bone-positive remodeling, demonstrating that 25 (OH)D3 deficiency did not impair biochemical markers of bone metabolism in this subset of patients [127,128].

In MM patients, a recommended daily dose of 400 IU of VitD is now considered inadequate, while a higher daily dose of 1000 IU seems to be optimal for promoting bone health and improving outcomes through maintenance, after the correction of deficits [125,129]. Deficit states of vitD require higher doses of about 1500–2000 IU/day [130].

It was recently reported by Yellapragad et al. that the correlation between vitamin D deficiency and a poor MM prognosis that occurs in white patients does not occur in black patients, revealing a biological difference in MM between different ethnic groups [8], with better survival in African American patients [131]. A weaker correlation of VitD levels with bone mineral density, resulting in a lower fracture risk, has also been demonstrated in black patients, which is probably due to an abundance of the vitamin D-binding protein [132,133]. 

### Vitamin D Status and MGUS/SMM Risk Progression

VitD also plays a key role in patients with monoclonal gammopathy of undetermined significance (MGUS) and smoldering multiple myeloma (SMM) through the dysregulation of RANKL (receptor activator of nuclear factor kB ligand) and OPG (osteoprotegrin) [129,134]. Thus, the risk of progression associated with these conditions is increased in patients deficient in VitD. 

Several studies have reported skeletal changes in patients with MGUS such as increased cortical porosity, and reduced bone mineral density and trabecular and cortical thickness [135,136]. These changes occur irrespective of the M protein levels, which are related to an increased risk of fractures compared to the healthy population, especially of the spine [137,138]. In MGUS patients, these alterations seem to be encouraged by an increase in the levels of osteoclast-activating factors, such as CCL3/MIP-1a (chemokine ligand 3/macrophage inflammatory protein 1-alpha) and RANKL/OPG, along with an increase in DKK1 (Dickkopf-related protein 1), an osteoblast-suppressive factor with higher gene expression in MGUS plasma cells compared with controls [135]. 

It is well known that patients with MGUS residing at latitudes distant from the equator experience higher rates of progression due to the reduced UVB irradiance [139,140,141]. Diet is also partly responsible for vitamin deficiency, and a poor diet increases the risk of developing symptomatic myeloma [142]. Following these findings, some researchers examined the correlation between serum VitD levels of 50 MGUS and SMM patients and markers of bone metabolism including RANKL and OPG, serum protein electrophoresis (SPEP), and free light chains (FLCs). They then categorized patients into two risk of progression categories: low or intermediate-1 risk, and intermediate-2 or high risk. They showed that oral calciferol supplementation (6000 I.U. daily for 8 weeks followed by 2000 I.U. daily) is correlated with a reduction in bone disease progression [143]. 

**Figure 2:**
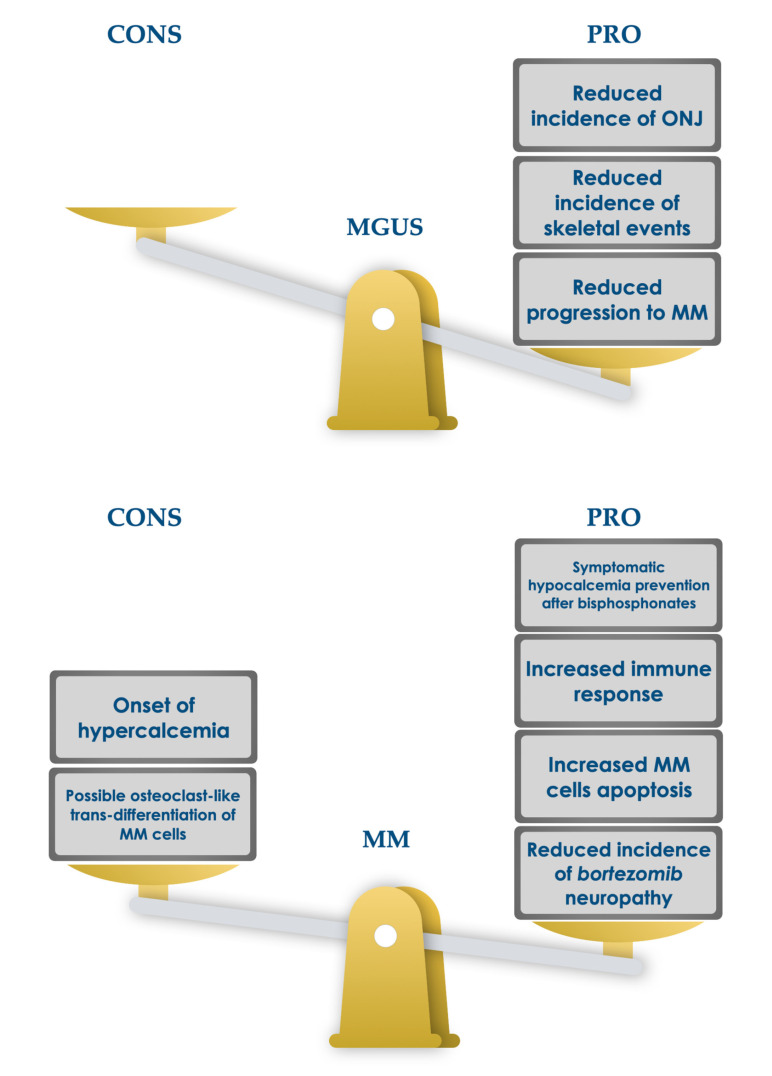
Possible unfavorable and favorable effects of vitamin D administration in MGUS subjects and MM patients [144,145,146,147,148,149,150].

Despite the importance of calcium and vitamin D in patients with MGUS, assessment practices are yet to be standardized, unlike symptomatic myeloma patients [91]. Given our results, these parameters should form an element of the flow chart of essential data required for the evaluation of asymptomatic patients, and any deficiency should be supported with oral compounds to maintain calcium homeostasis and bone health. Although the administration of alendronate does not conform to current guidelines for MM treatment, in an experimental study, a combination of oral alendronate (70 mg/week) and 1000 mg/daily calcium plus 880 IU/day of VitD revealed a reduction in lumbar fractures at 18 months in MGUS, with or without osteoporosis, compared with untreated patients [144,145]. This supplementation therapy also reduced SREs in SMM patients, although without showing a significant reduction in the progression rates of symptomatic MM [146]. In any case, the prophylactic use of bisphosphonates is not yet supported by good safety profiles (e.g., a high risk of osteonecrosis of the jaw, musculoskeletal pain, esophageal cancer, ocular inflammation, atrial fibrillation, oversuppression of bone turnover, and subtrochanteric femoral fractures) [147,148]. However, a 2009 study reported the ability of 1.25 (OH)2 D3 (10 nM), together with PDGF (platelet-derived growth factor) and PTH, to reduce, if not avoid, ONJ in chronical bisphosphonate-treated patients, thus inducing osteoblastic cell differentiation, proliferation, and viability [149]. On the other hand, the immunomodulating activity of vitD could play an important role in the immune-mediated hematological progression of patients with MGUS (Figure 2) [150]. 

Although the immune system has been implicated in the development of symptomatic MM, the scientific literature on the role and status of various immune components in this process is sometimes contradictory [151,152]. However, these assumptions have supported the development of drugs with powerful immunomodulatory activities, which are now widely used, against MM cells [153]. The combined use of vitD and these drugs could have a synergistic effect on monoclonal gammopathies.

## 4. Conclusions

The use of increasingly effective drugs, with considerably better toxicity profiles than those of traditional chemotherapeutic drugs, has improved the outcomes and survival in a variety of tumor patients. Despite still being considered an incurable neoplasm, multiple myeloma represents a paradigm of disease chronicization. This is the result of the considerable progress made, especially with regard to the effectiveness of immunomodulating agents and monoclonal antibodies, which has improved thanks to the best management and support therapies. While bisphosphonates and denosumab are widely standardized in patients with SREs, the role of vitamin D has not been fully clarified, especially the optimal dose. In some cases, the outdated use of VitD even results in tissue toxicity, which can cause kidney damage [154]. There are many practical problems and questions that remain to be resolved. For instance, it is important to determine whether the VitD level should be evaluated before starting a treatment with bisphosphonates or denosumab. Likewise, we need to decide whether the dosage should be equal for patients with or without deficiency who are undergoing treatment with bisphospohonates or denosumab. 

We believe that a dosage of VitD concentrations is appropriate in all patients suffering from MM, in particular those undergoing treatment with anti-resorptive drugs. It has been established that all adults who are vitamin D deficient should be treated with 50,000 IU of vitamin D2 or vitamin D3 once a week for 8 weeks, or its equivalent of 6000 IU of vitamin D2 or vitamin D3 daily to achieve a blood level of 25(OH)D above 30 ng mL^–1^, followed by maintenance therapy of 1500–2000 IU d^–^Moreover, for patients at risk of VitD deficiency, adults aged 19–50 years-old require at least 600 IU/d of vitamin D to maximize bone health and muscle function [155]. Such dosages may be inadequate for patients being treated with anti-resorptive drugs. Alongside the known cases of hypocalcemia induced by bisphosphonates, in the literature, there are numerous cases of hypocalcemia after administration of denosumab in the context of severe VitD deficiency, and an early supplementation plays an important role in the prevention and management of hypocalcemia [131,156]. Finally, further studies are needed to ascertain whether the different mechanisms of action of denosumab do not necessitate a different prophylactic dosage of VitD than for bisphosphonates (400 I.U.).

On the basis of what has been reported, we consider it appropriate to suggest a vitamin D treatment in MM patients at a dose of 1000 IU/day, with higher doses needed to correct severe deficiencies. This review also aims to provide a basis for the treatment of asymptomatic patients with MGUS/SMM and moderate or severe vitamin D deficiency, who are known to suffer from a high-risk progression of symptomatic MM and the development of vertebral fractures.

## Data Availability

Not applicable.
